# Cumulus Cells Gene Expression Profiling in Terms of Oocyte Maturity in Controlled Ovarian Hyperstimulation Using GnRH Agonist or GnRH Antagonist

**DOI:** 10.1371/journal.pone.0047106

**Published:** 2012-10-17

**Authors:** Rok Devjak, Klementina Fon Tacer, Peter Juvan, Irma Virant Klun, Damjana Rozman, Eda Vrtačnik Bokal

**Affiliations:** 1 Division of Reproductive Medicine, Department of Obstetrics and Gynecology, University Medical Centre Ljubljana, Ljubljana, Slovenia; 2 Centre for Functional Genomics and Bio-Chips, Institute of Biochemistry, Faculty of Medicine, University of Ljubljana, Ljubljana, Slovenia; 3 Institute for Hygiene and Pathology of Animal Nutrition, Veterinary Faculty, University of Ljubljana, Ljubljana, Slovenia; Michigan State University, United States of America

## Abstract

In *in vitro* fertilization (IVF) cycles controlled ovarian hyperstimulation (COH) is established by gonadotropins in combination with gonadotropin-releasing hormone (GnRH) agonists or antagonists, to prevent premature luteinizing hormone (LH) surge. The aim of our study was to improve the understanding of gene expression profile of cumulus cells (CC) in terms of ovarian stimulation protocol and oocyte maturity. We applied Affymetrix gene expression profiling in CC of oocytes at different maturation stages using either GnRH agonists or GnRH antagonists. Two analyses were performed: the first involved CC of immature metaphase I (MI) and mature metaphase II (MII) oocytes where 359 genes were differentially expressed, and the second involved the two GnRH analogues where no differentially expressed genes were observed at the entire transcriptome level. A further analysis of 359 differentially genes was performed, focusing on anti-Müllerian hormone receptor 2 (*AMHR2*), follicle stimulating hormone receptor (*FSHR*), vascular endothelial growth factor C (*VEGFC*) and serine protease inhibitor E2 (*SERPINE2*). Among other differentially expressed genes we observed a marked number of new genes connected to cell adhesion and neurotransmitters such as dopamine, glycine and γ-Aminobutyric acid (GABA). No differential expression in CC between the two GnRH analogues supports the findings of clinical studies where no significant difference in live birth rates between both GnRH analogues has been proven.

## Introduction

*In vitro* fertilization (IVF) has become one of the most common treatments of infertility. In an IVF cycle, ovarian stimulation is established by gonadotropins in combination with gonadotropin-releasing hormone (GnRH) analogues, i.e. GnRH agonists or GnRH antagonists. GnRH analogues are used to prevent premature luteinizing hormone (LH) surge during ovarian stimulation, which improves oocyte yield and increases pregnancy rate [1]. In the 1980s a long protocol of GnRH agonists was used starting in the midluteal phase of the preceding cycle [2]. In the 1990s, GnRH antagonists were introduced into clinical practice and proved to be safe and effective [3], [4], [5]. In contrast to GnRH agonists, GnRH antagonists cause immediate and rapid gonadotropin suppression without an initial period of gonadotropin hypersecretion. GnRH antagonists have several advantageous effects over GnRH agonists [6], [7], of which the most important is having fewer follicles and lower oestradiol level on the day of human chorionic gonadotropin (hCG) application [4] leading to a lower incidence of ovarian hyperstimulation syndrome (OHSS) [8], a serious complication of assisted reproductive therapy. Further, with a shorter period of application GnRH antagonists are friendlier to patients. Earlier studies have shown that GnRH antagonists result in lower pregnancy and delivery rates compared to GnRH agonists [6], whereas recent meta analyses show that the difference between them is not significant [9], [10].

Despite great improvements in assisted reproductive technology the success of IVF still remains relatively low. Most of the oocytes retrieved after ovarian stimulation are capable of fertilization; however, only half of them develop into embryos and only a few implant. Therefore, more than one embryo is usually transferred to increase the pregnancy rate, which leads to multiple pregnancies, and increased fetal and maternal morbidity and mortality [11]. For the development of high quality embryos the maturity and quality of oocytes is fundamental. At present, oocyte competence is estimated only on the basis of morphological evaluation of the polar body, meiotic spindle, zona pellucida and cytoplasm. There is increasing evidence that morphological evaluation is not a reliable predictor of oocyte competence and embryo implantation potential. The development of functional genomics technologies has made more objective measures available such as gene expression in cumulus cells (CC) as a non-invasive prognostic indicator of oocyte fertilization competence [12], [13].

Cumulus cells are essential for oocytes development. During folliculogenesis, an intense bidirectional communication exists between oocytes and surrounding CC [14], which is crucial for the development of mature and competent oocytes. Consequently, CC may reflect oocyte quality and can be used for oocyte selection. The oocyte itself also plays an active role by secreting paracrine factors that maintain the appropriate microenvironment for the acquisition of its developmental competence [15], [16]. The oocyte-secreted paracrine factors influence gene expression and protein synthesis in granulosa cells (GC) and CC that in turn regulate oocyte developmental competence. Consequently, GC and CC can serve as indirect markers of oocyte quality. In IVF procedures, GC and CC are separated from oocytes and discarded, which is why they are easily accessible and also suitable for gene expression analysis of oocyte maturity [15].

Therefore, we used transcription profiling to perform two analyses: the first was focused on oocyte maturity and the second on the type of ovarian stimulation protocol used: recombinant gondadotropins in combination with either GnRH agonists or GnRH antagonists. The aim of this study was to improve the understanding of the CC gene expression profile in terms of ovarian stimulation protocol. To our knowledge this is the first assessment of both GnRH analogues at the molecular level in a prospective study.

## Materials and Methods

### Patients and IVF treatment

In this prospective, randomized study, 21 patients undergoing classical IVF cycle at the Department of Obstetrics and Gynecology, University Medical Center Ljubljana, were included. The study was approved by the national medical ethics committee and all patients have signed informed consent. Randomization was performed according to JL Fleiss [17]; the randomization list was prepared in advance. Each patient who agreed to participate in the study was assigned to either a GnRH agonist or a GnRH antagonist group, and had an equal chance to be assigned to either group. The allocation was carried out by revealing a therapy group by a third person to medical staff and the patient at the moment of entering the study. The inclusion criteria were as follows: age less than 35 years, body mass index (BMI) ranging between 17 and 26 kg/m2, indication for IVF program was tubal factor infertility, and the partner's spermiogram had to be normal according to WHO criteria.

Eleven randomly selected patients were administered GnRH agonist buserelin acetate (Suprefact; Hoechst AG, Frankfurt/Main, Germany) from day 22 at a daily dose of 0.6 ml (600 pg) subcutaneously. When the criteria for ovarian desensitization were fulfilled (oestradiol <0.05 nmol/L, follicles <5 mm in diameter), they were subcutaneously administered 225 IU of gonadotrophin folitropin α (Gonal F; Industria Farmaceutica Serono S.p.A, Bari, Italy). To the remaining 10 patients 225 IU of gonadotrophin follitropin α was subcutaneously administered on day 2. When the dominant follicle measured ≥14 mm in diameter, GnRH antagonist cetrorelix acetate (Cetrotide; Asta Medica AG, Frankfurt, Germany) in a dose of 0.25 mg was administered subcutaneously.

When at least three follicles were ≥17 mm and serum oestradiol was ≥0.40 nmol/L per follicle all patients were administered 10,000 IU of human chorionic gonadotrophin (hCG) (Pregnyl; N.V. Organon); 34–36 h later an ultrasound guided transvaginal oocyte retrieval was performed.

### Cumulus cell collection and oocyte follow up

Oocytes were removed from the follicular fluid. Immediately after oocyte retrieval, CC of each oocyte were removed by a needle and a glass denudation pipette (Swemed, Sweden), washed in PBS, snap frozen in liquid nitrogen and stored at −80°C in vials until RNA isolation.

The oocytes were further inseminated (classical IVF) and cultured individually. After 24 hours, oocyte fertilization status was assessed. Fertilized oocytes expressed two pronuclei and two polar bodies, whereas unfertilized oocytes did not. All unfertilized cells were denudated to assess the maturation stage. Immature MI oocytes were round cells which did not extrude polar bodies and did not express germinal vesicles, whereas mature MII oocytes had a clearly visible and developed polar body. Only CC obtained from MI and unfertilized MII oocytes were considered for transcriptome analysis. Fertilized oocytes were further cultured to the blastocyst stage in the Universal IVF Medium followed by BlastAssist System (M1 and M2; Origio, Denmark) for five days. On day 5, at most two embryos at the blastocyst or morula stage were transferred into the uterus. Supernumerary blastocysts were cryopreserved. Only CC obtained from MII oocytes developed to blastocyst stage embryo were considered in transcriptome analysis.

### Experimental design

The difference between the two IVF cycle stimulation protocols using GnRH analogues was studied at three different levels of oocyte quality: metaphase I oocytes (MI), unfertilized metaphase II (MII) oocytes (MII-NF) and MII oocytes developed to blastocyst stage embryo (MII-BL). We performed two analyses: firstly we assessed differences in CC gene expression according to the oocyte stage achieved (MI versus MII), and secondly we assessed the differences in CC gene expression according to the GnRH analogue used. By comparing the CC MI and MII expression profiles we sought for gene markers linked to oocyte maturity.

**Figure 1:**
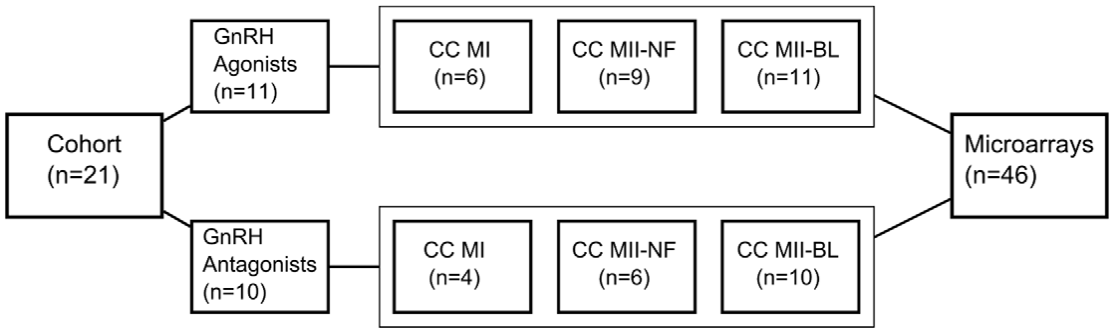
Experimental design. number of patients included in the study with respect to the two GnRH analogue treatments and the number of collected CC samples; CC MI: cumulus cells of metaphase I oocytes; CC MII-NF: cumulus cells of unfertilized metaphase II oocytes; CC MII-BL: cumulus cells of metaphase II oocytes developed to the blastocyst stage.

Figure 1 shows the number of patients (n = 21) and CC samples (n = 46) included in the study. Eleven patients were administered GnRH agonist and 10 GnRH antagonist. Altogether, 10 CC samples from MI oocytes, 15 from MII-NF oocytes and 21 from MII-BL oocytes were collected and considered in transcriptome analysis. Table 1 shows 21 patients randomized to either the GnRH agonist or GnRH antagonist group and the type and number of samples analyzed.

**Table 1:** A list of patients included in GnRH agonist and GnRH antagonist group with a number CC samples regarding to oocyte stage.

Patient	Therapy group	CC MI	CC MII-NF	CC MI-BL
1	GnRH agonist		1	1
2	GnRH agonist	1	1	1
3	GnRH agonist	1		1
4	GnRH agonist		1	1
5	GnRH agonist	1	1	1
6	GnRH agonist	1	1	1
7	GnRH agonist		1	1
8	GnRH agonist		1	1
9	GnRH agonist		1	1
10	GnRH agonist		2	1
11	GnRH agonist	1		1
12	GnRH antagonist		1	1
13	GnRH antagonist		1	1
14	GnRH antagonist		1	1
15	GnRH antagonist		1	1
16	GnRH antagonist	1		1
17	GnRH antagonist	1		1
18	GnRH antagonist	1		1
19	GnRH antagonist	1		1
20	GnRH antagonist		1	1
21	GnRH antagonist		1	1

CC MI: cumulus cells of metaphase I oocytes; CC MII-NF: cumulus cells of unfertilized metaphase II oocytes; CC MII-BL: cumulus cells of metaphase II oocytes developed to blastocyst stage embryo.

### RNA preparation

RNA was extracted using TRI reagent (Sigma – Aldrich, St. Louis, USA) according to slightly modified manufacturer's instruction. Due to small sample volume, glycogen was used as a carrier to increase RNA yield. Briefly, CC from individual cumulus – oocyte complexes were homogenized in 500 µL TRI reagent supplemented with 125 µg of glycogen (Ambion, Austin, USA). After 2 min incubation at room temperature, 100 µL chloroform was added and the sample was vortexed vigorously. RNA was precipitated with isopropanol from the aqueous phase and collected after 15 min centrifugation at 12,000× g and 4°C. RNA pellet was washed 3 times by 75% ethanol, dried and dissolved in 15 µL of RNAse free water. The integrity of the RNA samples was assessed on Agilent 2100 Bioanalyzer to assure high quality of total RNA; the RIN number was more than 7 for each sample.

### Transcriptome analysis

Transcriptome analysis was performed using the GeneChip Human Gene 1.0 ST Arrays (Affymetrix, Santa Clara, USA). The arrays were hybridized according to manufacturer's recommendations. Briefly, 200 ng of RNA was amplified and converted to cDNA using the WT Expression Kit (Ambion, Austin, USA). The resulting cDNA was fragmented and labeled using the GeneChip WT Terminal Labeling and Controls Kit (Affymetrix, Santa Clara, USA) and hybridized to the arrays for 16 hours. The arrays were washed using GeneChip Fluidics Station 450 according to manufacturer's recommendations, and scanned on the Affymetrix GeneChip Scanner 3000 7G. The images were analyzed and quality of data checked by Affymetrix GeneChip Expression Console software.

Data were processed and analyzed using different R/Bioconductor packages [18]. Data were normalized using the RMA algorithm from the XPS package. The raw and normalized gene expression data together with experimental information were deposited to Gene Expression Omnibus database (http://www.ncbi.nlm.nih.gov/geo) in compliance with MIAME standards, under series accession number GSE34230.

**Figure 2:**
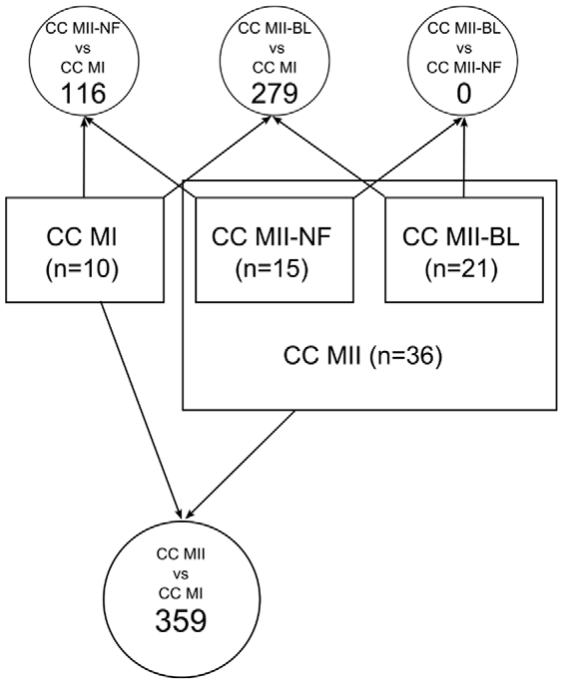
Schematic representation of microarray data analysis according to oocyte maturity. Boxes represent CC samples at different oocyte stages and their numbers; large box represents CC samples merged from MII-NF and MII-BL oocytes. Contrasts and corresponding numbers of differentially expressed genes are shown in circles. CC MI: cumulus cells of metaphase I oocytes; CC MII-NF: cumulus cells of unfertilized metaphase II oocytes; CC MII-BL: cumulus cells of metaphase II oocytes developed to the blastocyst stage; CC MII-NF vs. CC MI: contrast between CC MII-NF and CC MI; CC MII-BL vs. CC MI: contrast between CC MII-BL and CC MI; CC MII-BL vs. CC MII-NF: contrast between CC MII-BL and CC MII-NF; CC MII vs. CC MI: contrast between CC MII and CC MI.

**Figure 3:**
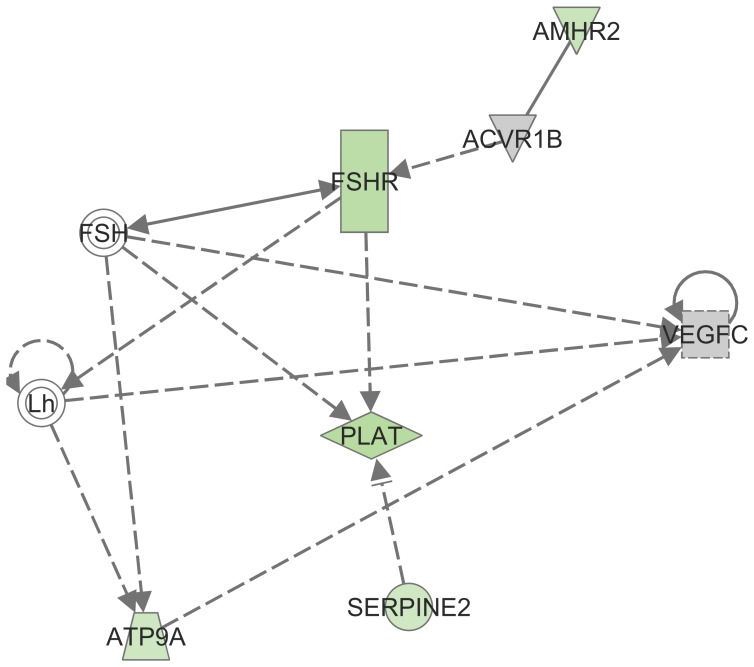
Subgroup of genes connected to follicle stimulating hormone (FSH) and luteinizing hormone (LH) from a top ranked gene network. It is associated with Post-Translational Modification, Cellular Development, Cellular Growth and Proliferation as identified by Ingenuity Pathways Analysis. Top ranked gene network was obtained from comparisons of differential expression between CC MII and CC MI. Genes are represented as nodes, and the biological relationship between two nodes is represented as edge (line): a plain line indicates direct interaction, a dashed line indicates indirect interaction; a line without arrowhead indicates binding only, a line finishing with a vertical line indicates inhibition; a line with an arrowhead indicates ‘acts on’. The green colour intensity of the nodes indicates the degree of down-regulation in the CC MII group. CC MI: cumulus cells of metaphase I oocytes. CC MII: cumulus cells of metaphase II oocytes.

The two-way ANOVA model was used to assess the differential expression of genes using LIMMA package by controlling the false discovery rate (FDR) [19] at significance level α = 0.05. Considered were 4 contrasts between different oocyte stages without considering the therapy (see Figure 2) and 3 contrasts between GnRH agonist and GnRH antagonist for each oocyte stage separately.

Annotation of genes and data representation was managed using ANNAFFY and AFFYCORETOOLS packages. Simultaneously, gene set enrichment analyses were performed using Parametric Gene Set Enrichment Analysis (PGSEA) package in combination with LIMMA package by controlling FDR at significance level α = 0.05. Gene sets related to different KEGG pathways were tested for their enrichment with respect to the contrasts of interest and sorted by their enrichment scores. Gene Ontology analysis was performed using GeneCodis [20]; a top ranked gene network among differentially expressed genes was identified by Ingenuity Pathway Analysis software (Ingenuity® Systems, www.ingenuity.com).

### Quantitative real time PCR (qPCR)

Quantitative real time PCR (qPCR) was used to validate 4 differentially expressed genes on a subset (30 out of 46) of samples using TaqMan Gene Expression pre-designed assays (Applied Biosystems, Foster City, USA). Genes for qPCR validation were selected considering their significance and their biological function in folliculogenesis. Cumulus cells samples are small and do not provide enough RNA to perform microarray analysis and qPCR validation in all samples. In our study 30 of the 46 samples provided enough RNA to perform both analyses. Peptidylprolyl isomerase B (*PPIB*) and 18s rRNA were added for normalization. Genomic DNA contamination was eliminated by DNAse treatment using DNAse I (Roche, Basel, Switzerland). cDNA for qPCR assays was prepared from 200 ng DNAsed RNA using SuperScript RT III (Invitrogen, Carlsbad, USA) in 20 µl final volume. Following cDNA synthesis, RNAse free water was added to increase the sample volume to 30 µl. The measurements were performed using LightCycler 480 System (Roche Applied Science, Penzberg, Germany). Normalized mRNA levels were obtained by dividing the averaged, efficiency corrected values for mRNA expression by normalization factor calculated from PPIB and 18s rRNA values and were expressed in arbitrary units [21]. The resulting values were log2 transformed (log2 fold change) for comparison with microarray data.

## Results

As patients were randomly assigned to either the GnRH agonist or GnRH antagonist group, we first assessed the baseline characteristics of the two treatment groups. The groups did not differ in age, BMI, pregnancy rate and delivery rate (Table 2). The number of retrieved oocytes was higher in the GnRH agonist group at a borderline significance level (p = 0.08). The proportions of degenerated, MI, MII, MII-NF and MII-BL oocytes were similar in both groups. The fertilization rate was by 30% higher in the GnRH agonist group (0.65 vs. 0.5; p = 0.06), which almost reached statistical significance.

**Table 2:** Patients baseline characteristics.

	GnRH agonists	GnRH antagonists	p-value
Age (years)	30.7±3.88	30.5±3.03	0.88
BMI (kg/m2)	23.1±2.68	22.7±2.80	0.72
Oestradiol level on day of hCG (nmol/L)	5.3±2.3	4.6±2.4	0.48
Retrieved oocytes (n)	11.7±5.24	8.0±3.89	0.08
Degenerated (ratio)	0.02±0.05	0.04±0.11	0.46
MI (proportion)	0.07±0.08	0.14±0.15	0.19
MII (proportion)	0.91±0.08	0.82±0.21	0.18
Fertilized (proportion)	0.65±0.14	0.50±0.19	0.06
MII-NF (proportion)	0.68±0.08	0.62±0.22	0.36
MII-BL (proportion)	0.23±0.08	0.20±0.07	0.31
Endometrial lining thickness (mm)	9.91±1.58	9.8±0.63	0.84
Pregnancy rate per cycle (proportion)	0.55±0.16	0.60±0.16	0.81
Delivery rate per cycle (proportion)	0.55±0.16	0.40±0.16	0.53
Frozen embryos remained (number)	0.70±0.95	0.40±0.70	0.43

age, body mass index (BMI), serum oestradiol level on day of hCG, number of retrieved oocytes; proportions of degenerated oocytes, metaphase I oocytes (MI), metaphase II oocytes (MII), fertilized oocytes, unfertilized metaphase II oocytes (MII-NF) and metaphase II oocytes developed to the blastocyst stage (MII-BL). Significance of differences between the two treatment groups was assessed using Student's t-test; two-tailed p-values are shown.

Pregnancy was assessed by measuring serum beta hCG value 14 days after hCG administration: if beta hCG was more than 40 IU/L, ultrasound examination was performed 2 weeks later. If gestational sac(s) were seen on ultrasound, the patient was considered pregnant, embryo heart rate confirmed a viable pregnancy. The pregnancy rate was calculated as a ratio of the number of pregnancies per cycle. The delivery rate was a ratio of the number of deliveries per cycle; all the babies were live born in this study. The miscarriage rate was 33% (n = 2) in the GnRH antagonist group, and 0% in the GnRH agonist group.

Further, we assessed the differential expression of genes with respect to various contrasts of interest. The contrasts between GnRH agonist and GnRH antagonist treatments exposed no differentially expressed genes according to the FDR-adjusted p-values. We did not observe any differentially expressed genes at the level of MI, MII-NF and MII-BL between the two GnRH analogues used.

### CC gene expression differences according to maturity stage of the oocyte

To identify the differences in gene expression in CC related to oocyte maturity, we compared the expression of CC MI, CC MII-NF and CC MII-BL regardless of the GnRH analogue used (Figure 2). One hundred and sixteen genes were differentially expressed between CC MII-NF and CC MI, 279 genes between CC MII-BL and CC MI, and none between CC MII-BL and CC MII-NF oocytes. This indicates that the main transcriptional changes in CC occur during oocyte transition from MI to MII stage; therefore we merged data from CC MII-BL and CC MII-NF samples and compared their expression to CC MI. The latter comparison yielded 359 differentially expressed genes (Table S1). Functional characterization of differentially expressed genes using PGSEA of KEGG pathways yielded enrichments of DNA replication, cell cycle, homologous recombination and p53 signaling pathway (Table S2). Gene Ontology analysis of 359 differentially expressed genes showed multicellular organismal development, signal transduction and cell adhesion to be top three gene groups (Table S3). The genes exposing the highest down and up regulation according to the fold change are shown in Table 3.

**Table 3:** Top down and up regulated genes between CC MII and CC MI.

Symbol	Description	p-value	Fold Change
*SFRP4*	secreted frizzled-related protein 4	<0.01	−5.0
*ITGB3*	integrin, beta 3 (platelet glycoprotein IIIa, antigen CD61)	<0.01	−3.3
*MGP*	matrix Gla protein	0.01	−3.1
*CRHBP*	corticotropin releasing hormone binding protein	0.04	−3.0
*BUB1*	budding uninhibited by benzimidazoles 1 homolog (yeast)	0.01	−2.7
*ANK2*	ankyrin 2, neuronal	<0.01	−2.5
*TSPAN7*	tetraspanin 7	0.02	−2.4
*TNFSF4*	tumor necrosis factor (ligand) superfamily, member 4	<0.01	−2.4
*PALLD*	palladin, cytoskeletal associated protein	<0.01	−2.2
*DSE*	dermatan sulfate epimerase	<0.01	−2.2
*CCDC99*	coiled-coil domain containing 99	<0.01	−2.2
*GPR63*	G protein-coupled receptor 63	<0.01	−2.2
*GLRA2*	glycine receptor, alpha 2	<0.01	−2.1
*BMP3*	bone morphogenetic protein 3	0.01	−2.1
*CDH3*	cadherin 3, type 1, P-cadherin (placental)	<0.01	−2.0
*FRMD4B*	FERM domain containing 4B	0.01	−2.0
*ID3*	inhibitor of DNA binding 3, dominant negative helix-loop-helix protein	0.01	−2.0
*NDP*	Norrie disease (pseudoglioma)	<0.01	−2.0
*GABRA5*	gamma-aminobutyric acid (GABA) A receptor, alpha 5	0.04	−2.0
*MAOB*	monoamine oxidase B	0.01	−2.0
*HSD11B1*	hydroxysteroid (11-beta) dehydrogenase 1	0.02	1.7
*PTGES*	prostaglandin E synthase	0.04	1.9
*SPOCK2*	sparc/osteonectin, cwcv and kazal-like domains proteoglycan (testican) 2	0.02	2.0
*C10orf10*	chromosome 10 open reading frame 10	<0.01	2.3
*NKAIN1*	Na+/K+ transporting ATPase interacting 1	<0.01	3.1

differentially expressed genes which expressed the highest down and up according to log2 fold change and p value<0.05. CC MI: cumulus cells of metaphase I oocytes, CC MII: cumulus cells of metaphase II oocytes.

Among 359 differentially expressed genes a top ranked gene network associated with Post-Translational Modification, Cellular Development, Cellular Growth and Proliferation was identified by Ingenuity Pathway Analysis (Figure S1). Among these genes there was also a subgroup of genes connected to follicle stimulating hormone (FSH) and LH which were further analysed by qPCR (Figure 3).

### Microarray data validation by qPCR

**Figure 4:**
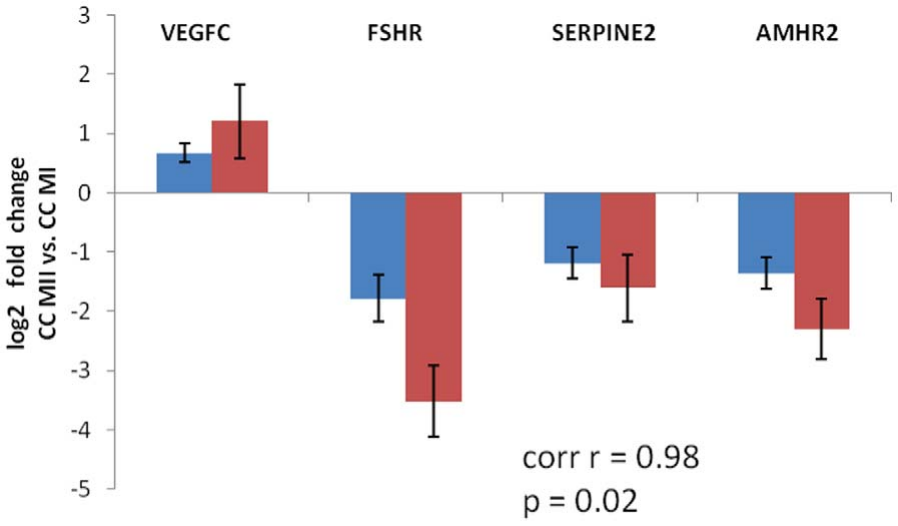
Expression (log2 fold change) of *VEGFC*, *SERPINE2*, *AMHR2* and *FSHR* from microarrays and qPCR showing contrast between CC MII and CC MI. Bars represent expression of CC MII vs. CC MI as log2 fold change with standard deviation. Blue represents microarray data and red qPCR data. Correlation coefficient (r = 0.98) and p value (p = 0.02) indicate strong and significant correlation between microarray and qPCR data. Corr r: Pearson correlation coefficient with p value between microarray and qPCR values. CC MI: cumulus cells of metaphase I oocytes. CC MII: cumulus cells of metaphase II oocytes.

Four of the 359 differentially expressed genes were validated by qPCR. Figure 4 shows the expression (log2 fold change) of anti-Müllerian hormone receptor 2 (*AMHR2*), follicle stimulating hormone receptor (*FSHR*), vascular endothelial growth factor C (*VEGFC*) and serine protease inhibitor E2 (*SERPINE2*) as assessed by microarray analysis and qPCR. All genes matched the direction of expression changes using either of the measurement method. Correlation factor (r) between log2 fold change of both methods for all the 4 genes was 0.98 (p = 0.02).

## Discussion

To our knowledge, this is the first prospective study comparing the effects of two different GnRH analogues and maturity stage of the oocyte at the level of gene expression in CC. Considering oocyte maturity we observed 359 differentially expressed genes between CC MI and CC MII. Using either GnRH agonists or GnRH antagonists, we have not observed differentially expressed genes. Moreover, we have not observed differentially expressed genes at any level of maturity stage of the oocyte (MI, MII-NF and MII-BL) between the two GnRH analogues used. Since only MII oocytes are capable of fertilization, our results support and supplement the clinical studies on GnRH analogues [9], [22] by showing that no significant differences between CC of MII oocytes exist at the transcriptome level. However, the GnRH antagonist protocol is more patient friendly because of shorter stimulation time, and is also considered safer because of a lower incidence of OHSS.

The analysis of baseline characteristics of the GnRH agonist and GnRH antagonist-treated patients showed that experimental groups were comparable, excluding sample-dependent biases in data. In line with previous findings [10], we have observed a higher number of retrieved oocytes and higher delivery rate in the GnRH agonist group although the differences were not significant. FDR corrected data analysis of CC transcriptome have shown that the two GnRH analogues do not differ significantly. A non detectable difference between GnRH agonists and GnRH antagonists at the CC transcriptional level is in accordance with clinical data considering pregnancy and delivery rates, showing slight and insignificant variances between the two GnRH analogues [9], [10], [22].

The analyses of gene expression in CC for each stage of oocyte maturity have shown that the most significant change at the transcriptional level occurs during oocyte transition from the MI to the MII stage. This is in accordance with previous studies [23], [24] reporting massive transcriptional changes in CC accompanied by a substantial transcript degradation in oocytes during the process of maturation [23]. We have found that the genes involved in the pathways of cell division, multicellular organismal development, signal transduction and cell adhesion play a significant role in the process of meiosis.

Among top down and up regulated genes comparing CC MI and CC MII there were previously described genes which are members of the Wnt signaling pathway (*SFRP4* and *CDH3*) [25], tumor growth factor beta (TGFβ) pathway (*BMP3*) [26], cell cycle (*BUB1*) [24] and prostaglandin formation (*PTGES*) [24], [27]. But the most numerous was a group of genes connected to cell adhesion, cytoskeleton and extracellular matrix formation [28] which included previously described genes (*ITGB3, ADAMTS1*) [29], [30] and many previously not described genes (*TSPAN7, SPOCK2*), and group of genes connected to signal transduction (*GLRA2, MAOB, GBRA5, GPR63*). These pathways have been previously recognised to have a key role in folliculogenesis and oocyte maturation [28], [31].

In the group of genes for extracellular matrix formation there were *TSPAN7*, which works through *ITGB3* [29], [32], MGP, which is a BMP2 binding protein [33]; they were among the top regulated genes between CC MI and CC MII. Further, a group of serpine peptidase inhibitors (*SERPINE2*, *SERPINF1*, *HTRA1*, *SERINC5*) with tissue plasminogen activator (*PLAT*) were also among downregulated genes. On the contrary, *SPOCK 2*, *NID2* and *ADAMTS1* showed upregulation between CC MI and CC MII oocytes. These genes are responsible for extracellular matrix binding. Extracellular matrix of CC has been found crucial for ovulation, oviduct passage and fertilization, especially through TNFIP6 protein [34]. The genes of CC extracellular matrix have been proved to be hCG-dependant [35]. *ANK2*, *ANK3* and *PALLD* are genes which function in cytoskeleton formation. All these genes were much less expressed in CC MII oocytes compared to CC MI oocytes. ANK2 and ANK3 both contain ANKRD57 protein, where the *ANKRD57* gene has already been recognized to be influenced by oocyte maturation in CC [36].

Among signal transduction genes other than Wnt signaling and TGFβ there were also top down and up regulated genes connected to neurotransmitters. Receptors for two inhibitory neurotransmitters glycine and γ-Aminobutyric acid GABA (*GLRA2*, *GABRA5*) and dopamine degrading enzyme gene (*MAOB*) were down regulated in CC MII compared to CC MI oocytes. So far little is known on the function of glycine and GABA in the folliculogenesis and oocyte maturation. A comparative analysis of amino acids in the follicular fluid of preovulatory follicles and in serum showed the amount of glycine differed the most, and was higher in the follicular fluid [37]. On the other hand more is known on the dopamine function in folliculogenesis. Dopamine derives from ovarian neurons in follicles [38] and during oocyte maturation its concentration rises [39]. From the follicular fluid dopamine is transported to the oocyte where it is degraded to noriepinephrine [38]. Another role of dopamine agonists is a blockage of VEGF vascular permeability in prevention of OHSS without compromising the result of IVF [40]. Considering the results of our study as well as of previous studies [37], [38], [40], [41] we suppose that neurotransmitters have an important role in folliculogenesis and oocyte maturation. To our knowledge this is the first implication that the genes responsive to dopamine, glycine and GABA might serve as biomarkers of oocyte maturation in CC.

The Ingenuity Pathway Analysis identified *AMHR2*, *FSHR*, *SERPINE2* and *VEGFC* to be in the top gene network among differentially expressed genes between CC MI and CC MII, and are connected to FSH and LH. The expression of *AMHR2* and *FSHR* in CC MI oocytes was significantly higher compared to CC MII oocytes. The expression of *FSHR* and *AMHR2* in CC has been reported to be strongly related to the expression level of anti-Müllerian hormone (*AMH*) and androgen receptor (*AR*) [42], [43], [44]. In the ovary, AMH regulates primordial follicle recruitment and FSH sensitivity of growing follicles in an inhibitory manner. *AMH* expression in GC and CC has been found to be the highest in pre-antral and small antral follicles [45], [46], and gradually diminishes during folliculogenesis [46], [47] which is in accordance with *AR*, *FSHR* and *AMHR2* mRNA expression [42]. *FSHR* is highly expressed in CC and is critical for oocyte maturation [48]. Its expression decreases along with the progression of maturation of bovine oocytes *in vitro* [49] and *in vivo* after hCG administration [49]. Catteau-Jonard *et al.* have found that *FSHR* together with *AMH*, *AMHR2*, and *AR* are overexpressed in GC from stimulated follicles of PCOS women indicating an oocyte maturation defect [50].

In addition, *SERPINE2* is significantly altered by oocyte stage. *SERPINE2* expression is increased by FSH [51] and is decreased after LH surge in GC of growing dominant bovine follicles [52], [53]. This goes in line with the results of our study where CC MII shows a lower expression of *SERPINE2* than CC MI. Finally, the expression difference of *VEGFC* has been observed between CC MI and CC MII level, where the latter have a significantly higher expression. Vascular endothelial growth factor (VEGF) is an angiogenic substance synthesized in theca cells and GC [54]. Structurally related to VEGF are also VEGFB and VEGFC [55] and they are all expressed in human GC [56]. It is known [57] that follicular VEGF concentrations are higher in preovulatory follicles compared to early antral follicles and that oocyte quality is related to the intrafollicular influence of VEGF [58]. VEGF concentrations in follicular aspirates containing MII oocytes that have fertilized are higher than of those containing MII oocytes that are not fertilized [58].

In this study the differences in gene expression have been observed between CC MI and CC MII. This is not only in accordance with previous studies, but also provides some new aspects of oocyte maturation at the molecular level. According to our CC gene expression profiling we may conclude that both treatment protocols of ovarian stimulation with either GnRH agonists or GnRH antagonists are equally effective. Herein we show that the expression of genes in CC of mature MII oocytes does not differ using either GnRH agonists or GnRH antagonists. Oocyte maturity is a prerequisite for oocyte fertilization and high-quality embryos. This goes well along with the findings of more recent meta analyses on GnRH analogues where no significant difference in live birth rates between both GnRH analogues has been proven [22].

## Supporting Information

Figure S1 **Top ranked gene network associated with Post-Translational Modification, Cellular Development, Cellular Growth and Proliferation as identified by Ingenuity Pathways Analysis.** Top ranked gene network was obtained from comparisons of differential expression between CC MII and CC MI. Genes are represented as nodes, and the biological relationship between two nodes is represented as edge (line): a plain line indicates direct interaction, a dashed line indicates indirect interaction; a line without arrowhead indicates binding only, a line finishing with a vertical line indicates inhibition; a line with an arrowhead indicates ‘acts on’. The green colour intensity of the nodes indicates the degree of down-regulation while the red colour intensity of the nodes indicates the degree of up-regulation in the CC MII group. CC MI: cumulus cells of metaphase I oocytes. CC MII: cumulus cells of metaphase II oocytes.
(EPS)

Table S1 **359 differentially expressed genes, p value, and their expression (log2 fold change) between CC MII and CC MI. CC MI: cumulus cells of metaphase I oocyte; CC MII: cumulus cells of metaphase II oocyte.**
(DOCX)

Table S2 **Top enriched KEGG pathways by PGSEA and their expression (log2 fold change) between CC MII and CC MI.** Significant (p<0.05) expression changes are shown in bold.
(DOCX)

Table S3 **Gene Ontology (GO) analysis performed by GeneCodis upon 359 differentially expressed genes.** Items: GO group; Items_Details: description of GO group; Hyp_c: corrected value of hypergeometrical test.
(DOCX)
