# Supplementary material for: Cumulus Cells Gene Expression Profiling in Terms of Oocyte Maturity in Controlled Ovarian Hyperstimulation Using GnRH Agonist or GnRH Antagonist
Source: PLoS One. 2012 Oct 17;7(10):e47106. doi: 10.1371/journal.pone.0047106 (PMC3474825; doi:10.1371/journal.pone.0047106)
Supplement: Table S1 — 359 differentially expressed genes, p value, and their expression (log2 fold change) between CC MII and CC MI. CC MI: cumulus cells of metaphase I oocyte; CC MII: cumulus cells of metaphase II oocyte. (DOCX) [file pone.0047106.s002.docx]

| **Symbol** | **Description** | **p-value** | **Fold Change** |
| --- | --- | --- | --- |
| *SFRP4* | secreted frizzled-related protein 4 | 0.00 | -5.04 |
| *ITGB3* | integrin, beta 3 (platelet glycoprotein IIIa, antigen CD61) | <0.01 | -3.3 |
| *MGP* | matrix Gla protein | 0.01 | -3.08 |
| *CRHBP* | corticotropin releasing hormone binding protein | 0.04 | -2.95 |
| *BUB1* | budding uninhibited by benzimidazoles 1 homolog (yeast) | 0.01 | -2.66 |
| *ANK2* | ankyrin 2, neuronal | <0.01 | -2.49 |
| *TSPAN7* | tetraspanin 7 | 0.02 | -2.42 |
| *TNFSF4* | tumor necrosis factor (ligand) superfamily, member 4 | <0.01 | -2.36 |
| *PALLD* | palladin, cytoskeletal associated protein | <0.01 | -2.23 |
| *CCDC99* | coiled-coil domain containing 99 | <0.01 | -2.2 |
| *DSE* | dermatan sulfate epimerase | <0.01 | -2.2 |
| *GPR63* | G protein-coupled receptor 63 | <0.01 | -2.18 |
| *GLRA2* | glycine receptor, alpha 2 | <0.01 | -2.14 |
| *BMP3* | bone morphogenetic protein 3 | 0.01 | -2.06 |
| *CDH3* | cadherin 3, type 1, P-cadherin (placental) | <0.01 | -2.01 |
| *FRMD4B* | FERM domain containing 4B | 0.01 | -1.99 |
| *ID3* | inhibitor of DNA binding 3, dominant negative helix-loop-helix protein | 0.01 | -1.98 |
| *NDP* | Norrie disease (pseudoglioma) | <0.01 | -1.98 |
| *GABRA5* | gamma-aminobutyric acid (GABA) A receptor, alpha 5 | 0.04 | -1.96 |
| *MAOB* | monoamine oxidase B | 0.01 | -1.95 |
| *KLHDC8A* | kelch domain containing 8A | 0.02 | -1.94 |
| *SPRR2B* | small proline-rich protein 2B | 0.02 | -1.94 |
| *LOC81691* | exonuclease NEF-sp | 0.02 | -1.93 |
| *PLAT* | plasminogen activator, tissue | <0.01 | -1.92 |
| *RALGAPA2* | Ral GTPase activating protein, alpha subunit 2 (catalytic) | <0.01 | -1.92 |
| *KLHL6* | kelch-like 6 (Drosophila) | 0.01 | -1.91 |
| *ALDH18A1* | aldehyde dehydrogenase 18 family, member A1 | <0.01 | -1.9 |
| *SERINC5* | serine incorporator 5 | 0.01 | -1.89 |
| *LAPTM4B* | lysosomal protein transmembrane 4 beta | 0.01 | -1.87 |
| *FAM19A2* | family with sequence similarity 19 (chemokine (C-C motif)-like), member A2 | <0.01 | -1.84 |
| *SLC26A2* | solute carrier family 26 (sulfate transporter), member 2 | <0.01 | -1.83 |
| *HTRA1* | HtrA serine peptidase 1 | 0.05 | -1.82 |
| *CDH6* | cadherin 6, type 2, K-cadherin (fetal kidney) | 0.01 | -1.8 |
| *NELL2* | NEL-like 2 (chicken) | 0.01 | -1.8 |
| *FSHR* | follicle stimulating hormone receptor | 0.01 | -1.78 |
| *STXBP5L* | syntaxin binding protein 5-like | 0.03 | -1.78 |
| *EXT1* | exostoses (multiple) 1 | 0.01 | -1.77 |
| *TNNI3* | troponin I type 3 (cardiac) | 0.02 | -1.77 |
| *ABLIM1* | actin binding LIM protein 1 | <0.01 | -1.73 |
| *WEE1* | WEE1 homolog (S. pombe) | 0.02 | -1.73 |
| *ZFPM2* | zinc finger protein, multitype 2 | 0.01 | -1.73 |
| *ITGB5* | integrin, beta 5 | <0.01 | -1.72 |
| *TMOD1* | tropomodulin 1 | <0.01 | -1.7 |
| *DSP* | desmoplakin | <0.01 | -1.66 |
| *DTL* | denticleless homolog (Drosophila) | <0.01 | -1.66 |
| *CHST1* | carbohydrate (keratan sulfate Gal-6) sulfotransferase 1 | <0.01 | -1.65 |
| *DRP2* | dystrophin related protein 2 | <0.01 | -1.65 |
| *SULT1C4* | sulfotransferase family, cytosolic, 1C, member 4 | 0.03 | -1.65 |
| *EPHX1* | epoxide hydrolase 1, microsomal (xenobiotic) | <0.01 | -1.64 |
| *VIT* | vitrin | <0.01 | -1.64 |
| *CACHD1* | cache domain containing 1 | <0.01 | -1.61 |
| *CEACAM21* | carcinoembryonic antigen-related cell adhesion molecule 21 | 0.01 | -1.61 |
| *GXYLT2* | glucoside xylosyltransferase 2 | <0.01 | -1.59 |
| *NDRG2* | NDRG family member 2 | <0.01 | -1.59 |
| *ZNF275* | zinc finger protein 275 | 0.01 | -1.58 |
| *DAPK1* | death-associated protein kinase 1 | <0.01 | -1.56 |
| *IFITM1* | interferon induced transmembrane protein 1 (9-27) | 0.04 | -1.56 |
| *OR7A5* | olfactory receptor, family 7, subfamily A, member 5 | 0.01 | -1.56 |
| *KLHL24* | kelch-like 24 (Drosophila) | 0.03 | -1.54 |
| *MRAP2* | melanocortin 2 receptor accessory protein 2 | 0.01 | -1.54 |
| *NRIP3* | nuclear receptor interacting protein 3 | 0.01 | -1.53 |
| *SYTL5* | synaptotagmin-like 5 | 0.01 | -1.52 |
| *SYNPO2* | synaptopodin 2 | 0.03 | -1.5 |
| *TDGF1* | teratocarcinoma-derived growth factor 1 | 0.01 | -1.5 |
| *PTGIS* | prostaglandin I2 (prostacyclin) synthase | 0.02 | -1.49 |
| *SLCO3A1* | solute carrier organic anion transporter family, member 3A1 | <0.01 | -1.48 |
| *TRANK1* | tetratricopeptide repeat and ankyrin repeat containing 1 | <0.01 | -1.47 |
| *SLC1A4* | solute carrier family 1 (glutamate/neutral amino acid transporter), member 4 | 0.01 | -1.44 |
| *COX18* | COX18 cytochrome c oxidase assembly homolog (S. cerevisiae) | 0.01 | -1.43 |
| *HSD17B11* | hydroxysteroid (17-beta) dehydrogenase 11 | 0.04 | -1.43 |
| *TSPAN6* | tetraspanin 6 | 0.02 | -1.43 |
| *ENPP1* | ectonucleotide pyrophosphatase/phosphodiesterase 1 | 0.01 | -1.42 |
| *GRIK2* | glutamate receptor, ionotropic, kainate 2 | 0.03 | -1.41 |
| *ST6GAL1* | ST6 beta-galactosamide alpha-2,6-sialyltranferase 1 | <0.01 | -1.41 |
| *SLC44A5* | solute carrier family 44, member 5 | 0.03 | -1.39 |
| *C17orf57* | chromosome 17 open reading frame 57 | <0.01 | -1.38 |
| *SEMA7A* | semaphorin 7A, GPI membrane anchor (John Milton Hagen blood group) | 0.01 | -1.38 |
| *DAG1* | dystroglycan 1 (dystrophin-associated glycoprotein 1) | 0.01 | -1.37 |
| *NOSTRIN* | nitric oxide synthase trafficker | 0.04 | -1.37 |
| *NRP2* | neuropilin 2 | 0.01 | -1.37 |
| *AMHR2* | anti-Mullerian hormone receptor, type II | <0.01 | -1.36 |
| *CYTSB* | cytospin B | <0.01 | -1.36 |
| *DAAM1* | dishevelled associated activator of morphogenesis 1 | 0.01 | -1.36 |
| *FIBIN* | fin bud initiation factor homolog (zebrafish) | <0.01 | -1.36 |
| *MLLT11* | myeloid/lymphoid or mixed-lineage leukemia (trithorax homolog, Drosophila); translocated to, 11 | 0.03 | -1.36 |
| *C10orf72* | chromosome 10 open reading frame 72 | <0.01 | -1.35 |
| *GOLPH3L* | golgi phosphoprotein 3-like | 0.01 | -1.35 |
| *EDNRB* | endothelin receptor type B | 0.02 | -1.34 |
| *GLB1L2* | galactosidase, beta 1-like 2 | 0.04 | -1.33 |
| *NAV1* | neuron navigator 1 | 0.01 | -1.33 |
| *NOX4* | NADPH oxidase 4 | 0.02 | -1.33 |
| *SERPINF1* | serpin peptidase inhibitor, clade F (alpha-2 antiplasmin, pigment epithelium derived factor), member 1 | 0.01 | -1.33 |
| *SPRR2A* | small proline-rich protein 2A | 0.03 | -1.33 |
| *AMIGO2* | adhesion molecule with Ig-like domain 2 | 0.01 | -1.31 |
| *UST* | uronyl-2-sulfotransferase | <0.01 | -1.31 |
| *GLIPR2* | GLI pathogenesis-related 2 | 0.01 | -1.29 |
| *HIST1H4L* | histone cluster 1, H4l | 0.03 | -1.29 |
| *NRK* | Nik related kinase | <0.01 | -1.29 |
| *PRICKLE1* | prickle homolog 1 (Drosophila) | 0.02 | -1.29 |
| *UBE2T* | ubiquitin-conjugating enzyme E2T (putative) | 0.02 | -1.29 |
| *LOC643763* | hypothetical LOC643763 | <0.01 | -1.28 |
| *IFI35* | interferon-induced protein 35 | <0.01 | -1.27 |
| *DDX60L* | DEAD (Asp-Glu-Ala-Asp) box polypeptide 60-like | 0.02 | -1.26 |
| *USP35* | ubiquitin specific peptidase 35 | 0.01 | -1.26 |
| *ARHGEF6* | Rac/Cdc42 guanine nucleotide exchange factor (GEF) 6 | 0.01 | -1.25 |
| *CTSL1* | cathepsin L1 | 0.02 | -1.25 |
| *MYO5B* | myosin VB | 0.02 | -1.25 |
| *DPF3* | D4, zinc and double PHD fingers, family 3 | <0.01 | -1.24 |
| *ANK3* | ankyrin 3, node of Ranvier (ankyrin G) | 0.01 | -1.23 |
| *ACAN* | aggrecan | 0.03 | -1.21 |
| *ARSB* | arylsulfatase B | 0.01 | -1.21 |
| *ATP9A* | ATPase, class II, type 9A | <0.01 | -1.21 |
| *MRAS* | muscle RAS oncogene homolog | <0.01 | -1.2 |
| *PDE1B* | phosphodiesterase 1B, calmodulin-dependent | <0.01 | -1.2 |
| *RDH10* | retinol dehydrogenase 10 (all-trans) | 0.01 | -1.2 |
| *H2AFY2* | H2A histone family, member Y2 | 0.01 | -1.19 |
| *MAMDC2* | MAM domain containing 2 | 0.04 | -1.19 |
| *SERPINE2* | serpin peptidase inhibitor, clade E (nexin, plasminogen activator inhibitor type 1), member 2 | 0.01 | -1.19 |
| *TLR4* | toll-like receptor 4 | <0.01 | -1.19 |
| *TMEM144* | transmembrane protein 144 | 0.01 | -1.19 |
| *DOCK2* | dedicator of cytokinesis 2 | <0.01 | -1.17 |
| *PCDH18* | protocadherin 18 | <0.01 | -1.17 |
| *STEAP4* | STEAP family member 4 | 0.02 | -1.17 |
| *CDK1* | cyclin-dependent kinase 1 | 0.03 | -1.16 |
| *KAL1* | Kallmann syndrome 1 sequence | 0.01 | -1.16 |
| *CCDC80* | coiled-coil domain containing 80 | <0.01 | -1.15 |
| *CHEK1* | CHK1 checkpoint homolog (S. pombe) | 0.01 | -1.15 |
| *FBLN2* | fibulin 2 | 0.02 | -1.15 |
| *SLC44A1* | solute carrier family 44, member 1 | 0.03 | -1.15 |
| *ENC1* | ectodermal-neural cortex (with BTB-like domain) | 0.01 | -1.14 |
| *HMGA2* | high mobility group AT-hook 2 | 0.02 | -1.13 |
| *PHGDH* | phosphoglycerate dehydrogenase | 0.01 | -1.13 |
| *SLC10A5* | solute carrier family 10 (sodium/bile acid cotransporter family), member 5 | 0.03 | -1.13 |
| *SORL1* | sortilin-related receptor, L(DLR class) A repeats-containing | 0.02 | -1.13 |
| *AADAT* | aminoadipate aminotransferase | <0.01 | -1.12 |
| *ADAMTS16* | ADAM metallopeptidase with thrombospondin type 1 motif, 16 | 0.02 | -1.12 |
| *FHL2* | four and a half LIM domains 2 | <0.01 | -1.12 |
| *BRIP1* | BRCA1 interacting protein C-terminal helicase 1 | 0.01 | -1.11 |
| *KIAA0101* | KIAA0101 | 0.01 | -1.11 |
| *MEX3B* | mex-3 homolog B (C. elegans) | <0.01 | -1.11 |
| *TDP1* | tyrosyl-DNA phosphodiesterase 1 | <0.01 | -1.11 |
| *MAGED2* | melanoma antigen family D, 2 | 0.04 | -1.1 |
| *ABAT* | 4-aminobutyrate aminotransferase | 0.01 | -1.09 |
| *APOBEC3B* | apolipoprotein B mRNA editing enzyme, catalytic polypeptide-like 3B | 0.04 | -1.09 |
| *GJA1* | gap junction protein, alpha 1, 43kDa | 0.03 | -1.09 |
| *IVNS1ABP* | influenza virus NS1A binding protein | 0.01 | -1.09 |
| *MCM7* | minichromosome maintenance complex component 7 | 0.01 | -1.09 |
| *DIXDC1* | DIX domain containing 1 | <0.01 | -1.07 |
| *EML5* | echinoderm microtubule associated protein like 5 | 0.01 | -1.07 |
| *PLS3* | plastin 3 | 0.04 | -1.07 |
| *PPA1* | pyrophosphatase (inorganic) 1 | 0.03 | -1.07 |
| *TMEM117* | transmembrane protein 117 | 0.01 | -1.07 |
| *UBXN8* | UBX domain protein 8 | 0.01 | -1.07 |
| *BTG2* | BTG family, member 2 | 0.05 | -1.06 |
| *NEXN* | nexilin (F actin binding protein) | 0.03 | -1.06 |
| *CDK6* | cyclin-dependent kinase 6 | 0.01 | -1.04 |
| *AGPS* | alkylglycerone phosphate synthase | 0.05 | -1.03 |
| *CMTM6* | CKLF-like MARVEL transmembrane domain containing 6 | 0.01 | -1.03 |
| *MFAP3L* | microfibrillar-associated protein 3-like | 0.01 | -1.03 |
| *CHN1* | chimerin (chimaerin) 1 | 0.01 | -1.02 |
| *LAYN* | layilin | 0.03 | -1.02 |
| *PAK3* | p21 protein (Cdc42/Rac)-activated kinase 3 | 0.02 | -1.02 |
| *RRM1* | ribonucleotide reductase M1 | 0.02 | -1.02 |
| *EZH2* | enhancer of zeste homolog 2 (Drosophila) | 0.01 | -1.01 |
| *L3MBTL3* | l(3)mbt-like 3 (Drosophila) | 0.01 | -1.01 |
| *HPGD* | hydroxyprostaglandin dehydrogenase 15-(NAD) | 0.01 | -0.99 |
| *IFI44L* | interferon-induced protein 44-like | 0.02 | -0.99 |
| *RAB31* | RAB31, member RAS oncogene family | 0.03 | -0.99 |
| *ARHGAP26* | Rho GTPase activating protein 26 | 0.02 | -0.98 |
| *DCBLD1* | discoidin, CUB and LCCL domain containing 1 | 0.01 | -0.97 |
| *MED13L* | mediator complex subunit 13-like | 0.02 | -0.97 |
| *GREB1* | growth regulation by estrogen in breast cancer 1 | 0.02 | -0.96 |
| *PIR* | pirin (iron-binding nuclear protein) | 0.01 | -0.96 |
| *RP1-199H16.1* | similar to OTTHUMP00000028720 | <0.01 | -0.96 |
| *UNC5B* | unc-5 homolog B (C. elegans) | 0.03 | -0.96 |
| *CYP3A5* | cytochrome P450, family 3, subfamily A, polypeptide 5 | 0.03 | -0.95 |
| *FRY* | furry homolog (Drosophila) | <0.01 | -0.95 |
| *ORAI2* | ORAI calcium release-activated calcium modulator 2 | 0.02 | -0.95 |
| *WIF1* | WNT inhibitory factor 1 | 0.03 | -0.95 |
| *EPB41L1* | erythrocyte membrane protein band 4.1-like 1 | 0.02 | -0.94 |
| *MCM5* | minichromosome maintenance complex component 5 | 0.01 | -0.94 |
| *NAV2* | neuron navigator 2 | 0.05 | -0.94 |
| *NTN4* | netrin 4 | 0.05 | -0.94 |
| *RNF19A* | ring finger protein 19A | 0.03 | -0.94 |
| *IQGAP2* | IQ motif containing GTPase activating protein 2 | 0.01 | -0.93 |
| *KIAA0922* | KIAA0922 | 0.01 | -0.93 |
| *LRRC49* | leucine rich repeat containing 49 | 0.02 | -0.93 |
| *GINS1* | GINS complex subunit 1 (Psf1 homolog) | 0.03 | -0.92 |
| *PAWR* | PRKC, apoptosis, WT1, regulator | 0.04 | -0.9 |
| *AGPAT4* | 1-acylglycerol-3-phosphate O-acyltransferase 4 (lysophosphatidic acid acyltransferase, delta) | <0.01 | -0.89 |
| *HMOX1* | heme oxygenase (decycling) 1 | 0.01 | -0.89 |
| *MBOAT1* | membrane bound O-acyltransferase domain containing 1 | 0.03 | -0.89 |
| *CRIM1* | cysteine rich transmembrane BMP regulator 1 (chordin-like) | 0.01 | -0.88 |
| *GPR1* | G protein-coupled receptor 1 | 0.05 | -0.88 |
| *CEP192* | centrosomal protein 192kDa | 0.02 | -0.87 |
| *CYYR1* | cysteine/tyrosine-rich 1 | 0.02 | -0.86 |
| *FZD4* | frizzled homolog 4 (Drosophila) | <0.01 | -0.86 |
| *PHLDB1* | pleckstrin homology-like domain, family B, member 1 | <0.01 | -0.86 |
| *PRC1* | protein regulator of cytokinesis 1 | 0.04 | -0.85 |
| *BCL2L11* | BCL2-like 11 (apoptosis facilitator) | 0.05 | -0.84 |
| *HELLS* | helicase, lymphoid-specific | 0.02 | -0.84 |
| *ZNF436* | zinc finger protein 436 | 0.01 | -0.84 |
| *PIK3R3* | phosphoinositide-3-kinase, regulatory subunit 3 (gamma) | 0.04 | -0.82 |
| *RNF182* | ring finger protein 182 | 0.04 | -0.82 |
| *SFRP1* | secreted frizzled-related protein 1 | 0.01 | -0.82 |
| *SLC12A6* | solute carrier family 12 (potassium/chloride transporters), member 6 | 0.03 | -0.82 |
| *WEE1* | WEE1 homolog (S. pombe) | 0.01 | -0.82 |
| *MAML3* | mastermind-like 3 (Drosophila) | 0.04 | -0.81 |
| *FAM169A* | family with sequence similarity 169, member A | 0.04 | -0.8 |
| *GLRB* | glycine receptor, beta | 0.01 | -0.8 |
| *SF3A3* | splicing factor 3a, subunit 3, 60kDa | 0.03 | -0.8 |
| *DENND5B* | DENN/MADD domain containing 5B | 0.02 | -0.79 |
| *GPR125* | G protein-coupled receptor 125 | 0.03 | -0.79 |
| *JUP* | junction plakoglobin | 0.03 | -0.79 |
| *SLC16A4* | solute carrier family 16, member 4 (monocarboxylic acid transporter 5) | 0.04 | -0.78 |
| *DMD* | dystrophin | 0.04 | -0.76 |
| *DPH5* | DPH5 homolog (S. cerevisiae) | 0.02 | -0.76 |
| *GPD1L* | glycerol-3-phosphate dehydrogenase 1-like | 0.02 | -0.76 |
| *ZNF195* | zinc finger protein 195 | 0.03 | -0.76 |
| *C3orf59* | chromosome 3 open reading frame 59 | 0.03 | -0.75 |
| *C6orf167* | chromosome 6 open reading frame 167 | 0.04 | -0.75 |
| *LRP6* | low density lipoprotein receptor-related protein 6 | 0.01 | -0.75 |
| *LRRC2* | leucine rich repeat containing 2 | 0.02 | -0.75 |
| *RCC2* | regulator of chromosome condensation 2 | 0.01 | -0.75 |
| *RFWD3* | ring finger and WD repeat domain 3 | 0.03 | -0.75 |
| *FANCI* | Fanconi anemia, complementation group I | 0.03 | -0.74 |
| *SEC61A2* | Sec61 alpha 2 subunit (S. cerevisiae) | 0.01 | -0.74 |
| *WDR76* | WD repeat domain 76 | 0.05 | -0.74 |
| *BLM* | Bloom syndrome, RecQ helicase-like | 0.03 | -0.72 |
| *FZD5* | frizzled homolog 5 (Drosophila) | 0.01 | -0.72 |
| *MTA3* | metastasis associated 1 family, member 3 | 0.03 | -0.72 |
| *CCDC50* | coiled-coil domain containing 50 | 0.01 | -0.71 |
| *CTSS* | cathepsin S | 0.04 | -0.71 |
| *KIAA1797* | KIAA1797 | 0.01 | -0.71 |
| *DBN1* | drebrin 1 | 0.03 | -0.7 |
| *FAM113B* | family with sequence similarity 113, member B | 0.03 | -0.7 |
| *TJP2* | tight junction protein 2 (zona occludens 2) | 0.04 | -0.7 |
| *SHQ1* | SHQ1 homolog (S. cerevisiae) | 0.03 | -0.69 |
| *DUSP19* | dual specificity phosphatase 19 | 0.04 | -0.68 |
| *RCBTB2* | regulator of chromosome condensation (RCC1) and BTB (POZ) domain containing protein 2 | <0.01 | -0.68 |
| *PHF16* | PHD finger protein 16 | 0.02 | -0.67 |
| *SLC25A14* | solute carrier family 25 (mitochondrial carrier, brain), member 14 | 0.03 | -0.67 |
| *TMEM48* | transmembrane protein 48 | 0.04 | -0.67 |
| *ARNT2* | aryl-hydrocarbon receptor nuclear translocator 2 | 0.02 | -0.65 |
| *ACOX2* | acyl-Coenzyme A oxidase 2, branched chain | 0.01 | -0.64 |
| *POLD3* | polymerase (DNA-directed), delta 3, accessory subunit | 0.04 | -0.64 |
| *LPXN* | leupaxin | 0.04 | -0.63 |
| *NCAPD3* | non-SMC condensin II complex, subunit D3 | 0.01 | -0.63 |
| *SLC36A1* | solute carrier family 36 (proton/amino acid symporter), member 1 | 0.04 | -0.63 |
| *PHTF1* | putative homeodomain transcription factor 1 | 0.03 | -0.62 |
| *CC2D2A* | coiled-coil and C2 domain containing 2A | 0.01 | -0.61 |
| *KIAA1217* | KIAA1217 | 0.03 | -0.6 |
| *WDSUB1* | WD repeat, sterile alpha motif and U-box domain containing 1 | 0.05 | -0.6 |
| *CNNM2* | cyclin M2 | 0.04 | -0.59 |
| *SLC29A3* | solute carrier family 29 (nucleoside transporters), member 3 | 0.04 | -0.59 |
| *TUBGCP3* | tubulin, gamma complex associated protein 3 | 0.04 | -0.59 |
| *EPDR1* | ependymin related protein 1 (zebrafish) | 0.04 | -0.58 |
| *PLK1S1* | polo-like kinase 1 substrate 1 | 0.01 | -0.58 |
| *TUFT1* | tuftelin 1 | 0.02 | -0.58 |
| *FUT8* | fucosyltransferase 8 (alpha (1,6) fucosyltransferase) | 0.04 | -0.57 |
| *GJC1* | gap junction protein, gamma 1, 45kDa | 0.04 | -0.53 |
| *TMC7* | transmembrane channel-like 7 | 0.04 | -0.53 |
| *CSRP2BP* | CSRP2 binding protein | 0.03 | -0.5 |
| *SNX5* | sorting nexin 5 | 0.04 | -0.48 |
| *MAX* | MYC associated factor X | 0.04 | -0.43 |
| *FAM123B* | family with sequence similarity 123B | 0.03 | -0.39 |
| *NRL* | neural retina leucine zipper | 0.05 | 0.48 |
| *NUDT16P* | nudix (nucleoside diphosphate linked moiety X)-type motif 16 pseudogene | 0.05 | 0.49 |
| *PTOV1* | prostate tumor overexpressed 1 | 0.04 | 0.53 |
| *ACVR1B* | activin A receptor, type IB | 0.05 | 0.55 |
| *BCAR1* | breast cancer anti-estrogen resistance 1 | 0.04 | 0.55 |
| *MT1E* | metallothionein 1E | 0.04 | 0.58 |
| *BACE2* | beta-site APP-cleaving enzyme 2 | 0.04 | 0.6 |
| *PTPN18* | protein tyrosine phosphatase, non-receptor type 18 (brain-derived) | 0.04 | 0.6 |
| *PHPT1* | phosphohistidine phosphatase 1 | 0.01 | 0.62 |
| *BOK* | BCL2-related ovarian killer | 0.03 | 0.63 |
| *RNU105B* | RNA, U105B small nucleolar | 0.01 | 0.63 |
| *FIBCD1* | fibrinogen C domain containing 1 | 0.03 | 0.64 |
| *GATA6* | GATA binding protein 6 | 0.01 | 0.64 |
| *TSEN34* | tRNA splicing endonuclease 34 homolog (S. cerevisiae) | 0.02 | 0.64 |
| *ANKRD29* | ankyrin repeat domain 29 | 0.02 | 0.65 |
| *AXIN2* | axin 2 | 0.01 | 0.65 |
| *IRS2* | insulin receptor substrate 2 | 0.01 | 0.65 |
| *TEAD4* | TEA domain family member 4 | 0.03 | 0.66 |
| *VEGFC* | vascular endothelial growth factor C | 0.02 | 0.67 |
| *MT1JP* | metallothionein 1J (pseudogene) | 0.02 | 0.68 |
| *SLC6A8* | solute carrier family 6 (neurotransmitter transporter, creatine), member 8 | 0.05 | 0.68 |
| *WNT3* | wingless-type MMTV integration site family, member 3 | 0.01 | 0.69 |
| *MFHAS1* | malignant fibrous histiocytoma amplified sequence 1 | 0.03 | 0.7 |
| *CEBPD* | CCAAT/enhancer binding protein (C/EBP), delta | 0.02 | 0.72 |
| *ERN1* | endoplasmic reticulum to nucleus signaling 1 | 0.01 | 0.72 |
| *C5orf62* | chromosome 5 open reading frame 62 | 0.02 | 0.73 |
| *ALOX15B* | arachidonate 15-lipoxygenase, type B | 0.01 | 0.74 |
| *GADD45B* | growth arrest and DNA-damage-inducible, beta | 0.05 | 0.74 |
| *RHOU* | ras homolog gene family, member U | 0.02 | 0.74 |
| *CYB5R3* | cytochrome b5 reductase 3 | 0.03 | 0.76 |
| *MAL* | mal, T-cell differentiation protein | 0.05 | 0.78 |
| *SH2B3* | SH2B adaptor protein 3 | 0.01 | 0.8 |
| *MEIS3* | Meis homeobox 3 | 0.01 | 0.81 |
| *ECE1* | endothelin converting enzyme 1 | 0.03 | 0.82 |
| *PDE4DIP* | phosphodiesterase 4D interacting protein | 0.05 | 0.82 |
| *CCDC134* | coiled-coil domain containing 134 | 0.03 | 0.83 |
| *FAM183B* | acyloxyacyl hydrolase (neutrophil) | 0.04 | 0.83 |
| *CORO6* | coronin 6 | 0.02 | 0.85 |
| *EFHD2* | EF-hand domain family, member D2 | 0.04 | 0.85 |
| *MBOAT7* | membrane bound O-acyltransferase domain containing 7 | 0.04 | 0.85 |
| *CELA2B* | chymotrypsin-like elastase family, member 2B | 0.03 | 0.86 |
| *ESYT2* | extended synaptotagmin-like protein 2 | 0.01 | 0.86 |
| *NUDT16* | nudix (nucleoside diphosphate linked moiety X)-type motif 16 | <0.01 | 0.87 |
| *FNDC4* | fibronectin type III domain containing 4 | 0.02 | 0.88 |
| *SBNO2* | strawberry notch homolog 2 (Drosophila) | 0.01 | 0.9 |
| *FBLN7* | fibulin 7 | 0.01 | 0.91 |
| *COL18A1* | collagen, type XVIII, alpha 1 | 0.04 | 0.92 |
| *RAMP1* | receptor (G protein-coupled) activity modifying protein 1 | 0.04 | 0.92 |
| *ADRB1* | adrenergic, beta-1-, receptor | <0.01 | 0.93 |
| *RASAL2* | RAS protein activator like 2 | 0.01 | 0.93 |
| *AOC2* | amine oxidase, copper containing 2 (retina-specific) | <0.01 | 0.94 |
| *OSGIN1* | oxidative stress induced growth inhibitor 1 | <0.01 | 0.96 |
| *ROR1* | receptor tyrosine kinase-like orphan receptor 1 | 0.03 | 0.96 |
| *SDK1* | sidekick homolog 1, cell adhesion molecule (chicken) | 0.01 | 0.97 |
| *KIAA1958* | KIAA1958 | <0.01 | 0.98 |
| *TBL1X* | transducin (beta)-like 1X-linked | 0.02 | 0.99 |
| *SLC16A3* | solute carrier family 16, member 3 (monocarboxylic acid transporter 4) | 0.01 | 1 |
| *CA12* | carbonic anhydrase XII | 0.04 | 1.05 |
| *IGF2BP2* | insulin-like growth factor 2 mRNA binding protein 2 | 0.01 | 1.05 |
| *MFSD10* | major facilitator superfamily domain containing 10 | 0.01 | 1.06 |
| *SCARB1* | scavenger receptor class B, member 1 | 0.02 | 1.08 |
| *PRKX* | protein kinase, X-linked | 0.01 | 1.1 |
| *DNAJA2* | DnaJ (Hsp40) homolog, subfamily A, member 2 | 0.03 | 1.13 |
| *PLXNB3* | plexin B3 | 0.04 | 1.13 |
| *BCL6* | B-cell CLL/lymphoma 6 | 0.02 | 1.16 |
| *CDK16* | cyclin-dependent kinase 16 | <0.01 | 1.16 |
| *CELA2A* | chymotrypsin-like elastase family, member 2A | 0.03 | 1.16 |
| *SAT1* | spermidine/spermine N1-acetyltransferase 1 | 0.05 | 1.18 |
| *PTPRN* | protein tyrosine phosphatase, receptor type, N | 0.05 | 1.21 |
| *GRAMD1A* | GRAM domain containing 1A | 0.03 | 1.22 |
| *RASD1* | RAS, dexamethasone-induced 1 | 0.03 | 1.26 |
| *SOCS1* | suppressor of cytokine signaling 1 | 0.01 | 1.27 |
| *VTN* | vitronectin | 0.03 | 1.3 |
| *NELF* | nasal embryonic LHRH factor | <0.01 | 1.31 |
| *SEC14L2* | SEC14-like 2 (S. cerevisiae) | 0.01 | 1.35 |
| *PDE7B* | phosphodiesterase 7B | 0.03 | 1.37 |
| *ACTN2* | actinin, alpha 2 | 0.03 | 1.4 |
| *FKBP11* | FK506 binding protein 11, 19 kDa | <0.01 | 1.4 |
| *LOC100131726* | HCC-related HCC-C11_v3 | 0.01 | 1.4 |
| *PLIN2* | perilipin 2 | 0.03 | 1.46 |
| *SCML1* | sex comb on midleg-like 1 (Drosophila) | 0.01 | 1.48 |
| *LCT* | lactase | 0.01 | 1.49 |
| *MGAT4A* | mannosyl (alpha-1,3-)-glycoprotein beta-1,4-N-acetylglucosaminyltransferase, isozyme A | 0.03 | 1.5 |
| *ADAMTS1* | ADAM metallopeptidase with thrombospondin type 1 motif, 1 | 0.03 | 1.52 |
| *ERRFI1* | ERBB receptor feedback inhibitor 1 | 0.01 | 1.54 |
| *COBLL1* | COBL-like 1 | 0.01 | 1.63 |
| *NPAS2* | neuronal PAS domain protein 2 | 0.03 | 1.66 |
| *NID2* | nidogen 2 (osteonidogen) | <0.01 | 1.67 |
| *HSD11B1* | hydroxysteroid (11-beta) dehydrogenase 1 | 0.02 | 1.74 |
| *PTGES* | prostaglandin E synthase | 0.04 | 1.94 |
| *SPOCK2* | sparc/osteonectin, cwcv and kazal-like domains proteoglycan (testican) 2 | 0.02 | 2 |
| *C10orf10* | chromosome 10 open reading frame 10 | <0.01 | 2.29 |
| *NKAIN1* | Na+/K+ transporting ATPase interacting 1 | <0.01 | 3.12 |
