# Supplementary material for: Cumulus Cells Gene Expression Profiling in Terms of Oocyte Maturity in Controlled Ovarian Hyperstimulation Using GnRH Agonist or GnRH Antagonist
Source: PLoS One. 2012 Oct 17;7(10):e47106. doi: 10.1371/journal.pone.0047106 (PMC3474825; doi:10.1371/journal.pone.0047106)
Supplement: Table S2 — Top enriched KEGG pathways by PGSEA and their expression (log2 fold change) between CC MII and CC MI. Significant (p<0.05) expression changes are shown in bold. (DOCX) [file pone.0047106.s003.docx]

| **Kegg pathway** | **p value** |
| --- | --- |
| DNA replication | **-1.21** |
| Cell cycle | **-1.72** |
| Homologous recombination | **-0.58** |
| p53 signaling pathway | **-0.85** |
