# Supplementary material for: Cumulus Cells Gene Expression Profiling in Terms of Oocyte Maturity in Controlled Ovarian Hyperstimulation Using GnRH Agonist or GnRH Antagonist
Source: PLoS One. 2012 Oct 17;7(10):e47106. doi: 10.1371/journal.pone.0047106 (PMC3474825; doi:10.1371/journal.pone.0047106)
Supplement: Table S3 — Gene Ontology (GO) analysis performed by GeneCodis upon 359 differentially expressed genes. Items: GO group; Items_Details: description of GO group; Hyp_c: corrected value of hypergeometrical test. (DOCX) [file pone.0047106.s004.docx]

| **Items** | **Items_Details** | **Hyp_c** |
| --- | --- | --- |
| GO:0007275 | multicellular organismal development (BP) | <0.01 |
| GO:0007165 | signal transduction (BP) | <0.01 |
| GO:0007155 | cell adhesion (BP) | <0.01 |
| GO:0008285 | negative regulation of cell proliferation (BP) | <0.01 |
| GO:0043065 | positive regulation of apoptotic process (BP) | <0.01 |
| GO:0060070 | canonical Wnt receptor signalling pathway | <0.01 |
| GO:0007420 | brain development (BP) | <0.01 |
| GO:0007399 | nervous system development (BP) | <0.01 |
| GO:0006915 | apoptotic process (BP) | <0.01 |
| GO:0000278 | mitotic cell cycle (BP) | <0.01 |
| GO:0007411 | axon guidance (BP) | <0.01 |
| GO:0007275,GO:0030154 | multicellular organismal development (BP),cell differentiation (BP) | <0.01 |
| GO:0045892 | negative regulation of transcription, DNA-dependent (BP) | <0.01 |
| GO:0051301 | cell division (BP) | <0.01 |
| GO:0030154 | cell differentiation (BP) | <0.01 |
| GO:0045944 | positive regulation of transcription from RNA polymerase II promoter (BP) | <0.01 |
| GO:0042493 | response to drug (BP) | <0.01 |
| GO:0006508 | proteolysis (BP) | <0.01 |
| GO:0006811 | ion transport (BP) | <0.01 |
| GO:0055085 | transmembrane transport (BP) | <0.01 |
| GO:0007596 | blood coagulation (BP) | <0.01 |
| GO:0045893 | positive regulation of transcription, DNA-dependent (BP) | <0.01 |
| GO:0000122 | negative regulation of transcription from RNA polymerase II promoter (BP) | 0.01 |
| GO:0006810 | transport (BP) | 0.05 |
